# Genomic Epidemiology of Clonal Complex 1 *Staphylococcus aureu*s in Remote Western Australian Communities

**DOI:** 10.1155/ijm/3445177

**Published:** 2025-11-19

**Authors:** Nicholas Wei Tek Yee, Geoffrey Wallace Coombs, Marc Stegger, Sharmin Baig, Hui-Leen Tan, Shakeel Mowlaboccus

**Affiliations:** ^1^School of Medical, Molecular and Forensic Sciences, Murdoch University, Perth, Western Australia, Australia; ^2^Department of Microbiology, PathWest Laboratory Medicine-WA, Fiona Stanley Hospital, Perth, Western Australia, Australia; ^3^Department of Sequencing and Bioinformatics, Statens Serum Institut, Copenhagen, Denmark; ^4^Harry Butler Institute, Murdoch University, Perth, Western Australia, Australia; ^5^School of Biomedical Sciences, The University of Western Australia, Perth, Western Australia, Australia

## Abstract

Community-associated methicillin-resistant *Staphylococcus aureus* (CA-MRSA) was first reported in Western Australia (WA) in the 1990s. Although ST8-IVa [2B] (WA-5) was the first identified CA-MRSA in WA, ST1-IVa [2B] (WA-1) soon emerged as the dominant clone. To investigate the genomic epidemiology of clonal complex (CC) CC1 *S. aureus* in WA Aboriginal communities from 1995 to 2003 and assess the acquisition and diversity of the SCC*mec* element, whole genome sequencing was performed. Three sequence types (STs) were identified: ST1 (81.4%), ST761 (0.9%) and ST762 (17.8%). MRSA constituted 78% (*n* = 92) of the collection and all harboured SCC*mec* Type IVa [2B]. Panton–Valentine leukocidin (PVL)–encoding genes were identified in seven closely related isolates. The phylogenetic tree topology suggests the acquisition of the same SCC*mec* IV into the CC1 lineage occurred on two occasions. Bayesian coalescence analysis predicts the CC1 *S. aureus* lineage originated in WA more than 150 years ago. Dissemination of the CC1 *S. aureus* lineage, as well as the horizontal acquisition of SCC*mec* IV, may have been aided by the concurrent movement of Aboriginal inhabitants across different remote communities.

## 1. Introduction

Methicillin-resistant *Staphylococcus aureus* (MRSA) produces a broad spectrum of clinical manifestations, ranging from skin and soft tissue infections to bacteraemia and infective endocarditis [1]. Although initially identified exclusively in the healthcare setting, community-associated methicillin-resistant *Staphylococcus aureus* (CA-MRSA) infections were identified in the late 1980s amongst Aboriginal people living in the remote communities located in the Kimberley region of Western Australia (WA). Using pulsed-field gel electrophoresis, Udo et al. identified four pulsotypes with the predominant pulsotype identified as multilocus Sequence Type (ST) 8 harbouring a Type IV staphylococcal cassette chromosome *mec* (SCC*mec*) element [2]. To determine the colonisation dynamics and genetics of CA-MRSA in the remote WA communities, epidemiological surveys were undertaken between 1995 and 2003 [3]. Of the 13 *S. aureus* clonal complexes (CCs) identified, five CCs contained methicillin-resistant isolates: CC1 (ST1-IVa [2B], colloquially known as WA-1), CC88 (ST78-IVa [2B], WA-2), CC5 (ST5-IVa [2B], WA-3), CC45 (ST45-V [5C2], WA-4) and CC8 (ST8-IVa [2B], WA-5). The five CA-MRSA clones were Panton–Valentine leukocidin (PVL)–negative, and ST1-IV (WA-1) predominated [3]. Subsequent to the O'Brien study, several studies have noted a heavy burden of staphylococcal disease and an increasing prevalence of CA-MRSA in Aboriginal people living across northern Australia [4]. In the Kimberley region, notification rates of CA-MRSA have significantly increased from 250 cases per 100,000 population in 2004 to 3625 cases per 100,000 in 2023 [5–7], with a disproportional number of cases associated with younger Aboriginal people. Furthermore, Aboriginal people infected with CA-MRSA are more likely to develop severe clinical manifestations compared to non-Aboriginal people [3, 8] and consequently require hospital services, particularly in the emergency department setting [4].

Although the population structure of CA-MRSA in remote WA is polyclonal, only a few clones have successfully adapted to the WA community environment [9]. The success of a CA-MRSA clone is often attributed to the clone harbouring a smaller SCC*mec* element (Type IV or V) and few antimicrobial resistance (AMR) or virulence genes. Although this is the case for WA-1, it is unclear if WA-1 is a single clone that has disseminated across the vast geographical landscape of WA or if ST1 methicillin-susceptible *S. aureus* (MSSA) clones have acquired the SCC*mec* on multiple occasions. In this study, using whole genome sequencing (WGS), we investigated the genomic epidemiology of the CC1 *S. aureus* isolated from the Aboriginal communities between 1995 and 2003 to determine if WA-1 CA-MRSA is a single clone.

## 2. Materials and Methods

### 2.1. Isolate Collection

**Figure 1:**
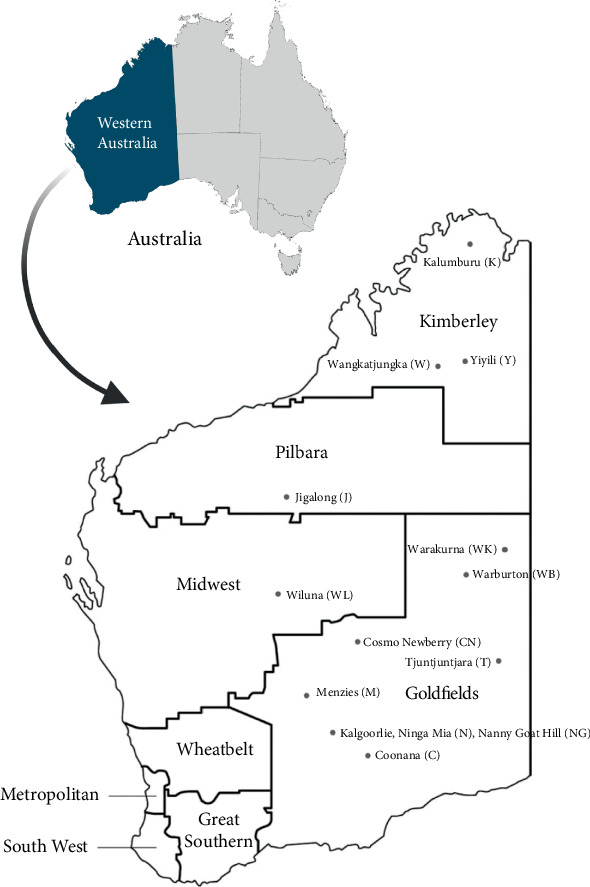
The geographic location of the 14 remote Aboriginal communities in Western Australia in respective health regions where the 137 Clonal Complex 1 (CC1) *S. aureus* isolates were isolated. Different communities with their respective abbreviations: C, Coonana; CN, Cosmo Newberry; J, Jigalong; K, Kalumburu; M, Menzies; NG, Nanny Goat Hill; N, Ninga Mia; T, Tjuntjuntjara; W, Wangkatjungka; WK, Warakurna; WB, Warburton; WL, Wiluna; Y, Yiyili. Different Western Australian public health regions: Goldfields, Great Southern, Kimberley, Metropolitan, Midwest, Pilbara, South West, Wheatbelt.

One hundred and thirty-seven CC1 *S. aureus* identified in the O'Brien et al. study were revived from −80°C 15% brain heart infusion glycerol broths or lyophilised tubes and subcultured twice on 5% sheep blood agar [3]. The isolates originated from 14 remote communities located in four WA health regions including the Kimberley (235,000 km^2^), Pilbara (508,000 km^2^), Midwest (605,000 km^2^) and Goldfields (950,000 km^2^) regions (Figure 1).

### 2.2. WGS

Genomic DNA was extracted using the MagMAX-96 DNA Multi-Sample Kit (Life Technologies, 4413021) and/or the DNeasy Blood & Tissue Kit (QIAGEN, United States, 69506) according to manufacturers' instructions. DNA quantification was performed using the Qubit 1X dsDNA HS Assay Kit (Thermo Fisher Scientific, Q33232). Sequencing libraries were prepared using the Illumina Nextera XT DNA Library Preparation Kit (Illumina, United States, FC-131-1096) and sequenced on the NextSeq 500 platform (Illumina, United States) with 150-bp paired-end chemistry.

### 2.3. Genomic Assembly and Bioinformatic Analyses

Raw sequence reads were assembled de novo using SPAdes v3.15.4 [10]. In silico multilocus sequence typing (MLST) was performed on genome assemblies to assign a ST and a CC to each isolate [11]. Genotyping methods, including SCC*mec* typing, staphylococcal protein A (*spa*) typing, accessory gene regulator (*agr*) typing and capsular serotyping, were performed on de novo assemblies using SCC*mec*Finder 1.2 and in-house bioinformatic pipelines [12]. The genomes were screened for toxin genes including the PVL-encoding genes (*lukF*-PV and *lukS*-PV) and the immune evasion cluster (IEC) genes (*sea*, *sep*, *sak*, *chp* and *scn*) using a BLAST interface [13]. AMR genetic determinants were identified using the AMRFinderPlus tool [14]. A single nucleotide polymorphism (SNP)–based analysis of the SCC*mec* IVa and SCC*fus* regions was performed using Snippy v4.6.0, with the CA05 composite SCC element (GenBank Accession No. AB063172.1) and MSSA476 (GenBank Accession No. BX571857.1) reference genome as references, respectively.

### 2.4. Phylogenetic Reconstruction

The CC1 *S. aureus* genomes were aligned to the MW2 USA400 ST1 reference genome (GenBank Accession No. NC_003923.1), and the alignment was used to construct a core SNP phylogeny using the maximum-likelihood algorithm and 100 bootstrap replicates in iTOL [15].

### 2.5. Coalescence-Based Analyses

Bayesian analysis was performed on BEAST v1.10.4 using Hasegawa–Kishino–Yano (HKY) and generalised time reversible (GTR) nucleotide substitution models, with gamma-distributed amongst-site variation with four rate categories to accommodate unequal base frequencies, transition and transversion bias. The models were combined with a strict molecular clock, or relaxed clock with different coalescent priors (constant-sized, logistic, exponential and expansion), constraining the distribution of time relative to genetic distance to determine the best substitution model [16]. The BEAGLE parallel computation library was also used to enhance the speed of likelihood calculations [17]. Evaluation of different model combinations was performed by determining the Bayes factor (BF) of each model. BF is the ratio of marginal likelihood estimates between two competing models [18]. The marginal likelihood estimate of each coalescent model was obtained from Tracer v.1.7.1 [19]. Two independent runs of 100 million iterations were performed for the selected model of choice based on the marginal likelihood estimate, and the chain was sampled every 10,000th generation. The runs containing Markov chain Monte Carlo (MCMC) samples were assessed for convergence and adequate (> 400) effective sample sizes (ESSs) for each parameter in Tracer v.1.7.1 [19]. The first 10% of each chain was discarded as burn-in. A maximum clade credibility (MCC) tree was generated to summarise the MCMC samples with TreeAnnotator v1.7.5. Visualisation and annotation of the MCC tree were performed in FigTree v1.4.4.

### 2.6. Phylogeographic Inference

Posterior probability estimates from the Bayesian analysis were visualised with the SpreaD3 v0.9.7 phylogeographic software [20]. A discrete phylogeographic map was created using the MCC tree, geographical coordinates of different communities and a geographical feature JavaScript Object Notation (GeoJSON) file of WA obtained from DataWA.

## 3. Results

### 3.1. Population Structure of CC1 Isolates

Overall, 118 of the 137 CC1 *S. aureus* were successfully revived and sequenced. Three *agr* Group III/capsular Serotype 8 CC1 STs were identified: ST1 [1-1-1-1-1-1-1] (*n* = 96), ST762 [1-1-104-1-1-1-1] (*n* = 21) and one ST761 [1-1-104-1-1-103-1] isolate. The ST761 and ST762 isolates were methicillin susceptible, whilst 92 (95.8%) of the ST1 isolates were methicillin resistant. Seven closely related *spa* types were identified: t127, t273, t559, t693, t2478, t10349 and t11670, with t127 (*n* = 84, 71.1%) the dominant *spa* type. The ST761 isolate and 19 of the 21 ST762 isolates were *spa* type t273 (Table S1).

### 3.2. Distribution of Virulence Genes

Two IEC types were identified in 116 (98.3%) of the isolates: Type D IEC (*sea*-*sak*-*scn*) was identified in 82 (70.7%) isolates and Type E IEC (*sak*-*scn*) in 34 (29.3%) isolates. The two remaining isolates had an intact *β*-haemolysin gene (*hlb*) and did not harbour IEC-associated genes. None of the isolates harboured the *chp* and *sep* IEC genes. The two PVL-encoding genes (*lukF*-PV and *lukS*-PV) were identified in seven ST1 MRSA isolates harbouring the *Φ*Sa2wa-st1 prophage (GenBank Accession No. MF580410.1). Four enterotoxin genes were identified (*sea*, *seh*, *sek* and *seq*) and at least one enterotoxin gene was detected in 116 (98.3%) isolates. The *seh* and *sek*+*seq* genes were identified in 110 (94.8%) and 96 (82.8%) isolates, respectively. Seventy-five (64.7%) isolates harboured all four enterotoxin genes. The exfoliative toxin A (*eta*) gene was identified in one ST762 isolate (Table S1).

### 3.3. Distribution of AMR Genes

All isolates harboured at least one AMR genetic determinant. Eight AMR genotypic markers associated with resistance to *β*-lactams (*blaZ*, *mecA*), chloramphenicol (*catA*), fusidic acid (*fusC*, H457Y FusC mutation), macrolides (*ermC*), quinolones (E84K ParC mutation) and tetracycline (*tet*(K)) were detected. The *blaZ* gene conferring penicillin resistance was identified in 114 (96.6%) isolates, and the *mecA* gene conferring methicillin resistance was identified in 92 (78.0%) isolates. The *catA* gene was identified in one (0.8%) isolate whilst the *ermC* gene was identified in 58 (49.2%) isolates. The *fusC* gene, identified in 64 (54.2%) isolates, was located within the SCC*fus* element. The 22.8 kb SCC*fus* element of all *fusC*-positive isolates had a pairwise nucleotide identity of 99.9%. The H457Y FusC and the E84K ParC mutations were each identified in a single isolate. The *tet*(K) gene was identified in three (2.5%) isolates (Table S1).

### 3.4. Phylogenetic Relatedness of CC1 Isolates

**Figure 2:**
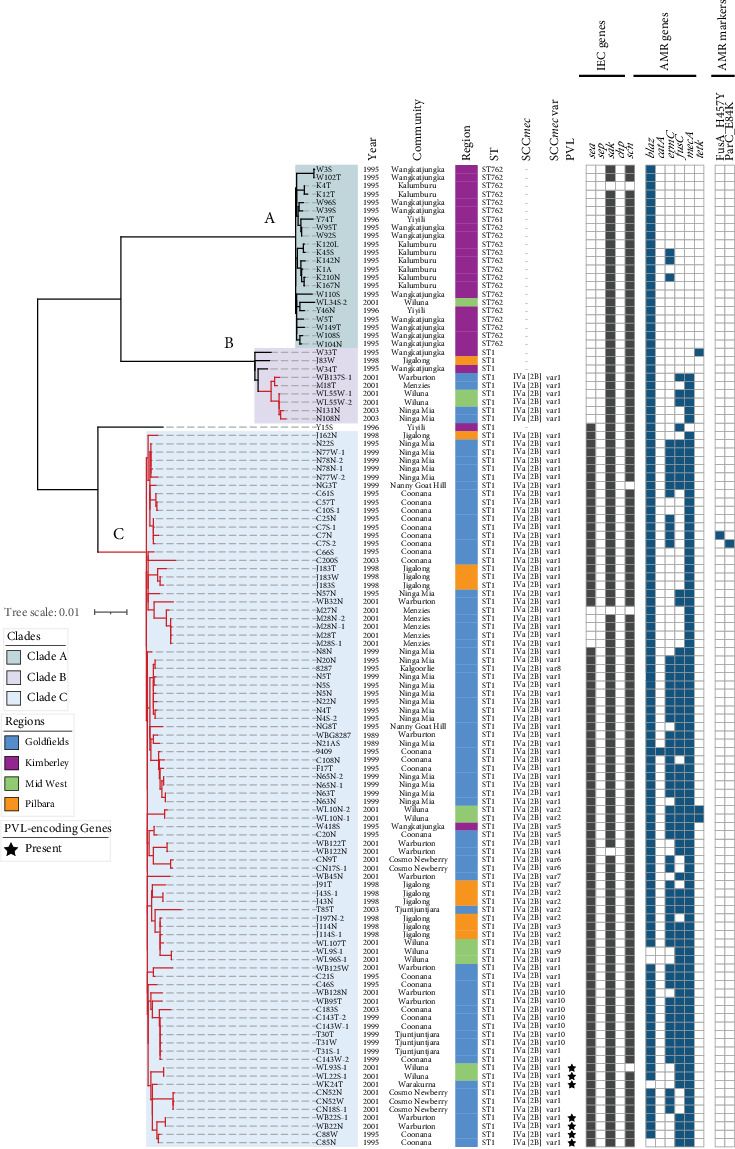
Maximum-likelihood phylogenetic tree of 118 Clonal Complex 1 (CC1) *S. aureus* isolates using the MW2 strain as reference (GenBank: NC_003923.1) based on 1629 core SNPs. Clades (A–C) are highlighted accordingly (A, green; B, purple; C, blue). The branch labels represent the isolate name, which consists of the community of origin, isolate ID and the type of specimen. Communities represented in the study: C, Coonana; CN, Cosmo Newberry; J, Jigalong; K, Kalumburu; M, Menzies; NG, Nanny Goat Hill; N, Ninga Mia; T, Tjuntjuntjara; W, Wangkatjungka; WK, Warakurna; WB, Warburton; WL, Wiluna; Y, Yiyili. Types of specimens: A, anterior nares; AS, anterior nares and skin; L, lesion; N, nasal; S, skin; T, throat; W, wound. Columns 1–5 represent the collection year, community of origin, respective health region, sequence type and the presence of SCC*mec* indicated by the subtype. Different SCC*mec* variants (var1–var10) identified are represented accordingly. The presence of Panton–Valentine leukocidin (PVL)–encoding genes (black star), immune evasion cluster (IEC) (grey square) and antimicrobial resistance (AMR) genes and markers (blue square) is indicated, respectively.

Based on the alignment of 1629 core SNPs, three phylogenetically distinct clades (Clades A, B and C) were identified (Figure 2). Except for one isolate (Y15S) which was an outlier, all isolates clustered in one of the three clades. Isolates in Clade A (*n* = 22) differed by 0–44 SNPs, Clade B isolates (*n* = 9) differed by 2–373 SNPs and Clade C isolates (*n* = 86) differed by 0–443 SNPs. Clade A contained the ST761 and ST762 MSSA isolates. Apart from one ST762 isolate, which was from the Midwest region, the Clade A isolates were from the Kimberley region. Clade B contained three ST1 MSSA and six ST1 MRSA isolates. The Clade B MSSA were from the Kimberley (*n* = 2) and Pilbara (*n* = 1) regions, whilst the Clade B MRSA were from the Goldfields (*n* = 4) and Midwest (*n* = 2) regions. All Clade C isolates were ST1 MRSA, 79.0% (*n* = 68) of which were from the Goldfields region whilst the remainder were from the Pilbara (*n* = 10), Midwest (*n* = 7) and Kimberley (*n* = 1) regions.

### 3.5. SCC*mec* Diversity

**Figure 3:**
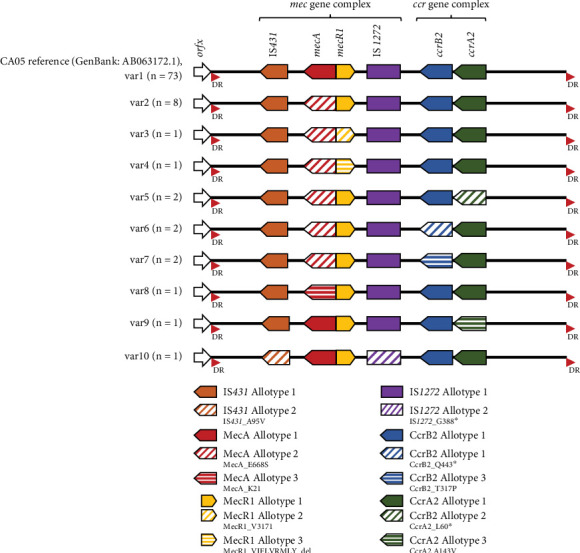
Schematic representation of different SCC*mec* IV [2B] element organisations in 92 Clonal Complex 1 (CC1) methicillin-resistant *S. aureus* (MRSA) isolates from Western Australia. The number of isolates harbouring each structural organisation (var1–var10) is shown accordingly. The allotypes of different structural elements are represented in different colours and patterns in the figure legend. The asterisk (∗) represents a stop codon. DR, direct repeat.

All 92 ST1 MRSA isolates harboured a 26.2-kb SCC*mec* IVa [2B] element with a pairwise identity of 99.3%. Based on nucleotide differences in the SCC*mec* IVa [2B] element, 10 variants (var1 to var10) were identified harbouring at least two allotypes of each structural element (IS*431*, *mecA*, *mecR1*, IS*1272*, *ccrB2* and *ccrA2*) (Figure 3). Seventy-three of the MRSA isolates (79.3%) harboured var1 which was identical to the structural elements harboured by the CA05 reference isolate. All Clade B isolates and 67 (77.9%) of Clade C isolates harboured var1.

### 3.6. Ancestral Dating and Phylogeographic Analysis

**Figure 4:**
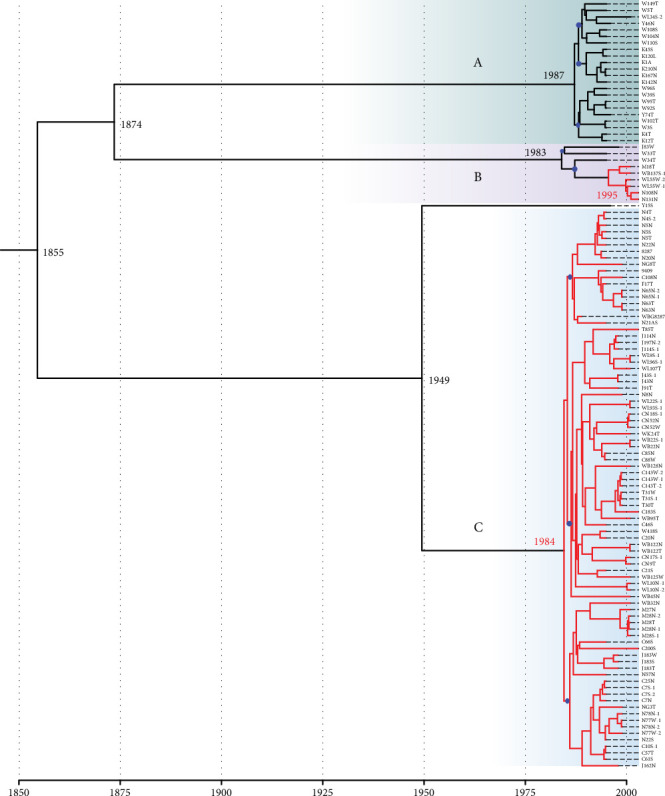
Bayesian maximum clade credibility tree of Clonal Complex 1 (CC1) *S. aureus* isolates from 1995 to 2003 using a strict clock model based on 1629 core SNPs. The branch tips are constrained by the year of isolation, and a timescale is represented at the bottom of the figure. Node branches containing CC1 methicillin-resistant *S. aureus* isolates are labelled in red. Clades (A–C) are highlighted accordingly (A, green; B, purple; C, light blue), and subclades are represented by a blue node.

**Figure 5:**
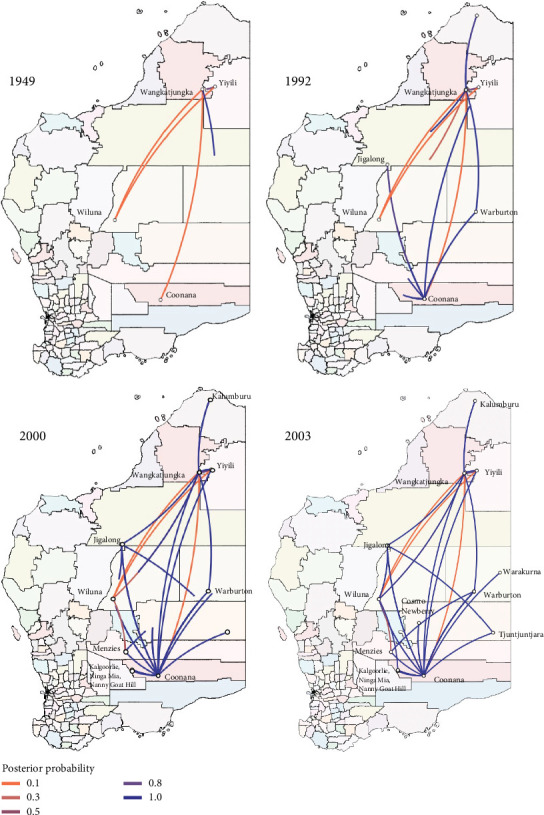
A phylogeographic map of the putative dissemination of Clonal Complex 1 (CC1) *S. aureus* strains in Western Australia across various time points (1949, 1992, 2000 and 2003). The white circles represent the communities where the inhabitants were sampled. The line colour indicates the level of posterior probability between different communities (0.1, orange; 1.0, purple). Various local government areas in Western Australia are coloured accordingly.

To determine when and how the CC1 isolates evolved and diverged over time, a coalescence analysis was performed in the identified SNP matrix. The best fitting Bayesian coalescent model was a GTR model with a strict molecular clock and constant population size. The prior for the clock rate was set to uniform (initial = 9.0 × 10^−4^, 0–1). The time to the most recent common ancestor (TMRCA) of the CC1 lineage was dated to 1855 (median: 1855, 95% highest posterior density [HPD]: 1824–1880), with a mean nucleotide substitution rate of 1.01 × 10^−3^ substitutions per site per year. Clades A and B are estimated to have separated in 1874 (95% HPD 1846–1897). Clade B diverged in 1983 (95% HPD 1979–1987), bifurcating into two subclades, whilst Clade A diverged later in 1987 (95% HPD 1985–1989) into three subclades. Clade C and the Y15S MSSA isolate separated in 1949 (95% HPD 1937–1959). Clade C diverged in 1984 (95% HPD 1982–1987) and resulted in three subclades. The acquisition of the SCC*mec* element within the CC1 lineage likely occurred on two occasions, first in 1984 (95% HPD 1982–1986) in Clade C and then in 1995 (95% HPD 1993–1997) in Clade B (Figure 4). Using a spatial phylogeographic analysis on the MCC tree, the concurrent movement of isolates across different communities was inferred. Lineage dissemination occurred frequently over 8 years, from 1992 to 2000. Communities with the most movement of strains included the Wangkatjungka and Coonana communities (Figure 5).

## 4. Discussion

Using WGS, we identified CC1 *S. aureus* isolated between 1995 and 2003 from Aboriginal populations living in remote WA communities consisted of three STs (ST1, ST761 and ST762), which harboured few AMR determinants. Apart from enterotoxin genes, few virulence genes were identified. Nucleotide similarity of the SCC*fus* element suggests the same SCC*fus* element has circulated amongst the CC1 isolates in WA since fusidic acid use for impetigo was frequent in the Kimberley region during the 1990s [21]. Bayesian phylogenetic analysis indicates that acquisition of the SCC*mec* element in Clades B and C occurred in 1984 (95% HPD 1982–1986) and 1995 (95% HPD 1993–1997), respectively. The exclusive presence of the SCC*mec* IVa [2B] element in ST1 isolates, along with the evidence that its acquisition occurred on two distinct occasions, supports the hypothesis of multiple acquisition events rather than a single acquisition leading to clonal expansion. Furthermore, the identification of var2 to var10 SCC*mec* IV suggests minor structural changes have occurred postacquisition in Clade C isolates during the 1990s. Despite evidence of movement between distantly remote Aboriginal communities, all Clade A isolates remained MSSA suggesting a mechanism that has prevented the acquisition of the SCC*mec* IV element [22]. Our findings suggest mutations in the *ccrAB* recombinase genes are likely the factor preventing SCC*mec* acquisition in the CC1 MSSA isolates.

Considering WA-1 is a dominant clone in the remote region of WA, it is notable that only a small proportion of ST1-MRSA harboured the PVL-encoding genes. Although PVL-positive ST93-IV and ST5-IV have become the dominant MRSA clone in WA, ST1-IV remains primarily PVL-negative [4, 7].

Although the remote communities are distantly apart—sometimes by more than several hundred kilometres, various anthropological studies have reported Aboriginal people living in the communities frequently travel across the country. Travel may be associated with visiting extended families, attending and conducting traditional ceremonies, hunting and accessing essential services such as schooling, banking and shopping [22, 23]. Movement between cattle stations in different communities, presumably for work-related reasons, also occurs. For example, inhabitants from the Wiluna community in the Midwest region travel to Jigalong in the Pilbara region (530 km away) and Warburton in the Goldfields (964 km away) to attend ritual ceremonies [24]. Similarly, inhabitants from the Tjuntjuntjara community frequently travel to Alice Springs in the Northern Territory (2900 km away) by car, stopping by other communities along the way [23]. The CC1 *S. aureus* lineage in WA disseminated over vast distances in a relatively short time period and was not restricted to one health region. Reconstruction of the spatial development of the CC1 isolates confirmed the concurrent transmission of isolates between communities and regions. The movement of Aboriginal people in the 1990s coincides with our evolutionary and phylogeographic analyses. Thus, the dissemination of CC1 MRSA was most likely due to the interaction of inhabitants from one community to the next community. The movement of inhabitants may also have contributed to the local dissemination of the WA-5 clone in the 1980s, leading to the identification of CA-MRSA strains with the same pulsotype across various isolated communities [2].

## 5. Conclusions

This study has elucidated the genomic epidemiology of the CC1 *S. aureus* lineage in WA between 1995 and 2003. The CC1 lineage harboured few AMR and virulence determinants and likely acquired the same SCC*mec* IVa [2B] element on more than one occasion. The widespread dissemination of the CC1 *S. aureus* lineage has been facilitated by the movement of Aboriginal people across different communities. Although WA-1 is now isolated throughout WA, the notification rates of CA-MRSA remain significantly higher for Aboriginal people compared to non-Aboriginal people. Findings from this study warrant the need for genomic surveillance on CA-MRSA to monitor circulating clones and to inform public health interventions.

## Data Availability

All data have been provided in the supporting information. More data can be provided on request.

## Supporting Information

Supporting Information Additional supporting information can be found online in the Supporting Information section. Table S1. The sequence type, *spa* type, virulence and antimicrobial resistance determinants of the *agr* III/capsular Serotype 8 Clonal Complex 1 (CC1) *S. aureus* identified in remote WA communities.

## References

[1] Lowy F. D. (1998). *Staphylococcus aureus* Infections. *New England Journal of Medicine*.

[2] Udo E. E., Pearman J. W., Grubb W. B. (1993). Genetic Analysis of Community Isolates of Methicillin-Resistant *Staphylococcus aureus* in Western Australia. *Journal of Hospital Infection*.

[3] O'Brien F. G., Coombs G. W., Pearman J. W. (2009). Population Dynamics of Methicillin-Susceptible and -Resistant *Staphylococcus aureus* in Remote Communities. *Journal of Antimicrobial Chemotherapy*.

[4] Bloomfield L. E., Coombs G., Armstrong P. (2024). Community-Associated Methicillin-Resistant *Staphylococcus aureus* in the Kimberley Region of Western Australia, Epidemiology and Burden on Hospitals. *Epidemiology and Infection*.

[5] Coombs G. W., Pearson J. C., Christiansen K. (2003). *Western Australian Antibiotic-Resistant Gram-Positive Bacteria Epidemiology and Typing Report (MRSA & VRE) 01 July -31- 31 December 2003*.

[6] Coombs G. W., Pearson J. C., Christiansen K. (2004). *Western Australian Antibiotic-Resistant Gram-Positive Bacteria Epidemiology and Typing Report (MRSA & VRE) 01 January -30 June 2004*.

[7] Coombs G. W., Tan H. L., Robinson J. O. (2023). *Western Australian Methicillin-Resistant Staphylococcus aureus (MRSA) Epidemiology and Typing Report: July 1 2022 to June 30 2023*.

[8] Munckhof W. J., Schooneveldt J., Coombs G. W., Hoare J., Nimmo G. R. (2003). Emergence of Community-Acquired Methicillin-Resistant *Staphylococcus aureus* (MRSA) Infection in Queensland, Australia. *International Journal of Infectious Diseases*.

[9] Coombs G. W., Monecke S., Pearson J. C. (2011). Evolution and Diversity of Community-Associated Methicillin-Resistant *Staphylococcus aureus* in a Geographical Region. *BMC Microbiology*.

[10] Prjibelski A., Antipov D., Meleshko D., Lapidus A., Korobeynikov A. (2020). Using SPAdes De Novo Assembler. *Current Protocols in Bioinformatics*.

[11] Jolley K. A., Bray J. E., Maiden M. C. J. (2018). Open-Access Bacterial Population Genomics: BIGSdb Software, the PubMLST.org Website and Their Applications. *Wellcome Open Research*.

[12] Kaya H., Hasman H., Larsen J. (2018). SCCmecFinder, a Web-Based Tool for Typing of Staphylococcal Cassette Chromosome *mec* in *Staphylococcus aureus* Using Whole-Genome Sequence Data. *mSphere*.

[13] Altschul S. F., Gish W., Miller W., Myers E. W., Lipman D. J. (1990). Basic Local Alignment Search Tool. *Journal of Molecular Biology*.

[14] Feldgarden M., Brover V., Gonzalez-Escalona N. (2021). AMRFinderPlus and the Reference Gene Catalog Facilitate Examination of the Genomic Links Among Antimicrobial Resistance, Stress Response, and Virulence. *Scientific Reports*.

[15] Letunic I., Bork P. (2021). Interactive Tree of Life (iTOL) v5: An Online Tool for Phylogenetic Tree Display and Annotation. *Nucleic Acids Research*.

[16] Drummond A. J., Suchard M. A., Xie D., Rambaut A. (2012). Bayesian Phylogenetics With BEAUti and the BEAST 1.7. *Molecular Biology and Evolution*.

[17] Ayres D. L., Cummings M. P., Baele G. (2019). BEAGLE 3: Improved Performance, Scaling, and Usability for a High-Performance Computing Library for Statistical Phylogenetics. *Systematic Biology*.

[18] Kass R. E., Raftery A. E. (1995). Bayes Factors. *Journal of the American Statistical Association*.

[19] Rambaut A., Drummond A. J., Xie D., Baele G., Suchard M. A. (2018). Posterior Summarization in Bayesian Phylogenetics Using Tracer 1.7. *Systematic Biology*.

[20] Bielejec F., Baele G., Vrancken B., Suchard M. A., Rambaut A., Lemey P. (2016). SpreaD3: Interactive Visualization of Spatiotemporal History and Trait Evolutionary Processes. *Molecular Biology and Evolution*.

[21] Bowen A. C., Daveson K., Anderson L., Tong S. Y. (2019). An Urgent Need for Antimicrobial Stewardship in Indigenous Rural and Remote Primary Health Care. *Medical Journal of Australia*.

[22] Young E., Kim D. (1989). *Mobility for Survival: A Process Analysis of Aboriginal Population Movement in Central Australia*.

[23] Peterson N. (2000). An Expanding Aboriginal Domain: Mobility and the Initiation Journey. *Oceania*.

[24] Sackett L. (1980). Working for the Law: Aspects of Economics in a Western Desert Community. *Anthropological Forum*.

