# Supplementary material for: Genomic Epidemiology of Clonal Complex 1 Staphylococcus aureus in Remote Western Australian Communities
Source: Int J Microbiol. 2025 Nov 19;2025:3445177. doi: 10.1155/ijm/3445177 (PMC12657077; doi:10.1155/ijm/3445177)
Supplement: Supporting Information — Additional supporting information can be found online in the Supporting Information section. Table S1. The sequence type, spa type, virulence and antimicrobial resistance determinants of the agr III/capsular Serotype 8 Clonal Complex 1 (CC1) S. aureus identified in remote WA communities. [file 3445177.f1.docx]

**Supplementary Table 1.** **The sequence type, *spa* type, virulence and antimicrobial resistance determinants of the *agr* III/capsular serotype 8 clonal complex 1 (CC1) *S. aureus* identified in remote WA communities.**

| Isolate | Region | Community | ST | *spa* type | IEC (Type) | PVL | Enterotoxins | Other virulence determinants | AMR  determinants |
| --- | --- | --- | --- | --- | --- | --- | --- | --- | --- |
| 8287 | Goldfields | Kalgoorlie | 1 | t127 | *sea*, *sak*, *scn* (D) | - | *sea, seh, sek+seq* | - | *blaZ, mecA, ermC, fusC* |
| 9409 | Goldfields | Coonana (C) | 1 | t127 | *sea*, *sak*, *scn* (D) | - | *sea, seh, sek+seq* | - | *blaZ, mecA, catA, ermC, fusC* |
| C108N | Goldfields | Coonana (C) | 1 | t127 | *sea*, *sak*, *scn* (D) | - | *sea, seh, sek+seq* | - | *blaZ, mecA, ermC* |
| C10S-1 | Goldfields | Coonana (C) | 1 | t127 | *sea*, *sak*, *scn* (D) | - | *sea, seh, sek+seq* | - | *blaZ, mecA* |
| C143T-2 | Goldfields | Coonana (C) | 1 | t127 | *sea*, *sak*, *scn* (D) | - | *sea, seh, sek+seq* | - | *blaZ, mecA, ermC, fusC* |
| C143W-1 | Goldfields | Coonana (C) | 1 | t127 | *sea*, *sak*, *scn* (D) | - | *sea, seh, sek+seq* | - | *blaZ, mecA, ermC, fusC* |
| C143W-2 | Goldfields | Coonana (C) | 1 | t127 | *sea*, *sak*, *scn* (D) | - | *sea, seh, sek+seq* | - | *blaZ, mecA, ermC, fusC* |
| C183S | Goldfields | Coonana (C) | 1 | t127 | *sea*, *sak*, *scn* (D) | - | *sea, seh, sek+seq* | - | *blaZ, mecA, ermC, fusC* |
| C200S | Goldfields | Coonana (C) | 1 | t127 | *sea*, *sak*, *scn* (D) | - | *sea, seh* | - | *blaZ, mecA* |
| C20N | Goldfields | Coonana (C) | 1 | t127 | *sea*, *sak*, *scn* (D) | - | *sea, seh, sek+seq* | - | *blaZ, mecA, ermC, fusC* |
| C21S | Goldfields | Coonana (C) | 1 | t127 | *sea*, *sak*, *scn* (D) | - | *sea, seh, sek+seq* | - | *blaZ, mecA, ermC, fusC* |
| C25N | Goldfields | Coonana (C) | 1 | t127 | *sea*, *sak*, *scn* (D) | - | *sea, seh, sek+seq* | - | *blaZ, mecA, ermC* |
| C46S | Goldfields | Coonana (C) | 1 | t127 | *sea*, *sak*, *scn* (D) | - | *sea, seh, sek+seq* | - | *blaZ, mecA, ermC, fusC* |
| C57T | Goldfields | Coonana (C) | 1 | t127 | *sea*, *sak*, *scn* (D) | - | *sea, seh, sek+seq* | - | *blaZ, mecA* |
| C61S | Goldfields | Coonana (C) | 1 | t127 | *sea*, *sak*, *scn* (D) | - | *sea, seh, sek+seq* | - | *blaZ, mecA, ermC* |
| C66S | Goldfields | Coonana (C) | 1 | t127 | *sea*, *sak*, *scn* (D) | - | *sea, seh, sek+seq* | - | *blaZ, mecA* |
| C7N | Goldfields | Coonana (C) | 1 | t127 | *sea*, *sak*, *scn* (D) | - | *sea, seh, sek+seq* | - | *blaZ, mecA, ermC,* FusA_H457Y mutation |
| C7S-1 | Goldfields | Coonana (C) | 1 | t127 | *sea*, *sak*, *scn* (D) | - | *sea, seh, sek+seq* | - | *blaZ, mecA, ermC* |
| C7S-2 | Goldfields | Coonana (C) | 1 | t127 | *sea*, *sak*, *scn* (D) | - | *sea, seh, sek+seq* | - | *blaZ, mecA, ermC,* ParC_E84K mutation |
| C85N | Goldfields | Coonana (C) | 1 | t127 | *sea*, *sak*, *scn* (D) | *lukF/S*-PV | *sea, seh, sek+seq* | - | *mecA, fusC* |
| C88W | Goldfields | Coonana (C) | 1 | t127 | *sea*, *sak*, *scn* (D) | *lukF/S*-PV | *sea, seh, sek+seq* | - | *blaZ, mecA, fusC* |
| CN17S-1 | Goldfields | Cosmo Newberry (CN) | 1 | t127 | *sea*, *sak*, *scn* (D) | - | *sea, sek+seq* | - | *blaZ, mecA, ermC* |
| CN18S-1 | Goldfields | Cosmo Newberry (CN) | 1 | t127 | *sea*, *sak*, *scn* (D) | - | *sea, seh, sek+seq* | - | *blaZ, mecA, ermC* |
| CN52N | Goldfields | Cosmo Newberry (CN) | 1 | t127 | *sea*, *sak*, *scn* (D) | - | *sea, seh, sek+seq* | - | *blaZ, mecA, ermC* |
| CN52W | Goldfields | Cosmo Newberry (CN) | 1 | t127 | *sea*, *sak*, *scn* (D) | - | *sea, seh, sek+seq* | - | *blaZ, mecA, ermC* |
| CN9T | Goldfields | Cosmo Newberry (CN) | 1 | t127 | *sea*, *sak*, *scn* (D) | - | *sea, seh, sek+seq* | - | *blaZ, mecA, ermC* |
| F17T | Goldfields | Coonana (C) | 1 | t127 | *sea*, *sak*, *scn* (D) | - | *sea, seh, sek+seq* | - | *blaZ, mecA, ermC, fusC* |
| M18T | Goldfields | Menzies (M) | 1 | t10349 | *sak*, *scn* (E) | - | *-* | - | *blaZ, mecA* |
| M27N | Goldfields | Menzies (M) | 1 | t127 | - | - | *seh* | - | *blaZ, mecA* |
| M28N-1 | Goldfields | Menzies (M) | 1 | t127 | *sak*, *scn* (E) | - | *seh, sek+seq* | - | *blaZ, mecA* |
| M28N-2 | Goldfields | Menzies (M) | 1 | t127 | *sak*, *scn* (E) | - | *seh, sek+seq* | - | *blaZ, mecA* |
| M28S-1 | Goldfields | Menzies (M) | 1 | t127 | *sak*, *scn* (E) | - | *seh, sek+seq* | - | *blaZ, mecA* |
| M28T | Goldfields | Menzies (M) | 1 | t127 | *sak*, *scn* (E) | - | *seh, sek+seq* | - | *blaZ, mecA* |
| N108N | Goldfields | Ninga Mia (N) | 1 | t11670 | *sak*, *scn* (E) | - | *seh* | - | *blaZ, mecA* |
| N131N | Goldfields | Ninga Mia (N) | 1 | t11670 | *sak*, *scn* (E) | - | *seh* | - | *blaZ, mecA* |
| N20N | Goldfields | Ninga Mia (N) | 1 | t127 | *sea*, *sak*, *scn* (D) | - | *sea, seh, sek+seq* | - | *blaZ, mecA, ermC, fusC* |
| N21AS | Goldfields | Ninga Mia (N) | 1 | t127 | *sea*, *sak*, *scn* (D) | - | *sea, seh, sek+seq* | - | *blaZ, mecA, ermC, fusC* |
| N22N | Goldfields | Ninga Mia (N) | 1 | t127 | *sea*, *sak*, *scn* (D) | - | *sea, seh, sek+seq* | - | *blaZ, mecA, ermC, fusC* |
| N22S | Goldfields | Ninga Mia (N) | 1 | t127 | *sea*, *sak*, *scn* (D) | - | *sea, seh, sek+seq* | - | *blaZ, mecA, ermC, fusC* |
| N4S-2 | Goldfields | Ninga Mia (N) | 1 | t127 | *sea*, *sak*, *scn* (D) | - | *sea, seh, sek+seq* | - | *blaZ, mecA, ermC, fusC* |
| N4T | Goldfields | Ninga Mia (N) | 1 | t127 | *sea*, *sak*, *scn* (D) | - | *sea, seh, sek+seq* | - | *blaZ, mecA, ermC, fusC* |
| N57N | Goldfields | Ninga Mia (N) | 1 | t127 | *sea*, *sak*, *scn* (D) | - | *sea* | - | *blaZ, mecA, fusC* |
| N5N | Goldfields | Ninga Mia (N) | 1 | t127 | *sea*, *sak*, *scn* (D) | - | *sea, seh, sek+seq* | - | *blaZ, mecA, ermC, fusC* |
| N5S | Goldfields | Ninga Mia (N) | 1 | t127 | *sea*, *sak*, *scn* (D) | - | *sea, seh, sek+seq* | - | *blaZ, mecA, ermC, fusC* |
| N5T | Goldfields | Ninga Mia (N) | 1 | t127 | *sea*, *sak*, *scn* (D) | - | *sea, seh, sek+seq* | - | *blaZ, mecA, ermC, fusC* |
| N63N | Goldfields | Ninga Mia (N) | 1 | t127 | *sea*, *sak*, *scn* (D) | - | *sea, seh, sek+seq* | - | *blaZ, mecA, fusC* |
| N63T | Goldfields | Ninga Mia (N) | 1 | t127 | *sea*, *sak*, *scn* (D) | - | *sea, seh, sek+seq* | - | *blaZ, mecA, ermC, fusC* |
| N65N-1 | Goldfields | Ninga Mia (N) | 1 | t127 | *sea*, *sak*, *scn* (D) | - | *sea, sek+seq* | - | *blaZ, mecA, ermC, fusC* |
| N65N-2 | Goldfields | Ninga Mia (N) | 1 | t127 | *sea*, *sak*, *scn* (D) | - | *sea, seh, sek+seq* | - | *blaZ, mecA, ermC, fusC* |
| N77W-1 | Goldfields | Ninga Mia (N) | 1 | t127 | *sea*, *sak*, *scn* (D) | - | *sea, seh, sek+seq* | - | *blaZ, mecA, ermC, fusC* |
| N77W-2 | Goldfields | Ninga Mia (N) | 1 | t127 | *sea*, *sak*, *scn* (D) | - | *sea, seh, sek+seq* | - | *blaZ, mecA, ermC, fusC* |
| N78N-1 | Goldfields | Ninga Mia (N) | 1 | t127 | *sea*, *sak*, *scn* (D) | - | *sea, seh, sek+seq* | - | *blaZ, mecA, ermC, fusC* |
| N78N-2 | Goldfields | Ninga Mia (N) | 1 | t127 | *sea*, *sak*, *scn* (D) | - | *sea, sek+seq* | - | *blaZ, mecA, ermC, fusC* |
| N8N | Goldfields | Ninga Mia (N) | 1 | t127 | *sea*, *sak*, *scn* (D) | - | *sea, seh, sek+seq* | - | *blaZ, mecA, fusC* |
| NG3T | Goldfields | Nanny Goat Hill (NG) | 1 | t127 | *sea*, *sak*, *scn* (D) | - | *sea, seh, sek+seq* | - | *blaZ, mecA, ermC, fusC* |
| NG8T | Goldfields | Nanny Goat Hill (NG) | 1 | t127 | *sea*, *sak*, *scn* (D) | - | *sea, seh, sek+seq* | - | *blaZ, mecA, fusC* |
| T30T | Goldfields | Tjuntjunjara (T) | 1 | t127 | *sea*, *sak*, *scn* (D) | - | *sea, seh, sek+seq* | - | *blaZ, mecA, ermC, fusC* |
| T31S-1 | Goldfields | Tjuntjunjara (T) | 1 | t127 | *sea*, *sak*, *scn* (D) | - | *sea, seh, sek+seq* | - | *blaZ, mecA, ermC, fusC* |
| T31W | Goldfields | Tjuntjunjara (T) | 1 | t127 | *sea*, *sak*, *scn* (D) | - | *sea, seh, sek+seq* | - | *blaZ, mecA, ermC, fusC* |
| T85T | Goldfields | Tjuntjunjara (T) | 1 | t127 | *sea*, *sak*, *scn* (D) | - | *sea, seh, sek+seq* | - | *blaZ, mecA, ermC, fusC* |
| WB122N | Goldfields | Warburton (WB) | 1 | t127 | *sea*, *sak*, *scn* (D) | - | *sea, seh, sek+seq* | - | *blaZ, mecA, fusC* |
| WB122T | Goldfields | Warburton (WB) | 1 | t127 | *sea*, *sak*, *scn* (D) | - | *sea, seh, sek+seq* | - | *blaZ, mecA, fusC* |
| WB125W | Goldfields | Warburton (WB) | 1 | t127 | *sea*, *sak*, *scn* (D) | - | *sea, seh, sek+seq* | - | *blaZ, mecA, ermC, fusC* |
| WB128N | Goldfields | Warburton (WB) | 1 | t127 | *sea*, *sak*, *scn* (D) | - | *sea, seh, sek+seq* | - | *blaZ, mecA, fusC* |
| WB137S-1 | Goldfields | Warburton (WB) | 1 | t10349 | *sak*, *scn* (E) | - | *seh* | - | *blaZ, mecA, fusC* |
| WB22N | Goldfields | Warburton (WB) | 1 | t127 | *sea*, *sak*, *scn* (D) | *lukF/S*-PV | *sea, seh, sek+seq* | - | *blaZ, mecA, fusC* |
| WB22S-1 | Goldfields | Warburton (WB) | 1 | t127 | *sea*, *sak*, *scn* (D) | *lukF/S*-PV | *sea, seh, sek+seq* | - | *blaZ, mecA, fusC* |
| WB32N | Goldfields | Warburton (WB) | 1 | t127 | *sea*, *sak*, *scn* (D) | - | *sea, seh, sek+seq* | - | *blaZ, mecA, fusC* |
| WB45N | Goldfields | Warburton (WB) | 1 | t127 | *sea*, *sak*, *scn* (D) | - | *sea, seh, sek+seq* | - | *blaZ, mecA, fusC* |
| WB95T | Goldfields | Warburton (WB) | 1 | t693 | *sea*, *sak*, *scn* (D) | - | *sea, seh, sek+seq* | - | *blaZ, mecA, ermC, fusC* |
| WBG8287 | Goldfields | Warburton (WB) | 1 | t127 | *sea*, *sak*, *scn* (D) | - | *sea, seh, sek+seq* | - | *blaZ, mecA, ermC, fusC* |
| WK24T | Goldfields | Warakurna (WK) | 1 | t127 | *sea*, *sak*, *scn* (D) | *lukF/S*-PV | *sea, sek+seq* | - | *mecA, fusC* |
| K120L | Kimberley | Kalumburu (K) | 762 | t127 | *sak*, *scn* (E) | - | *seh* | - | *blaZ* |
| K12T | Kimberley | Kalumburu (K) | 762 | t273 | *sak*, *scn* (E) | - | *seh* | - | *blaZ* |
| K142N | Kimberley | Kalumburu (K) | 762 | t273 | *sak*, *scn* (E) | - | *seh, sek+seq* | - | *blaZ, ermC* |
| K167N | Kimberley | Kalumburu (K) | 762 | t273 | *sak*, *scn* (E) | - | *seh* | - | *blaZ* |
| K1A | Kimberley | Kalumburu (K) | 762 | t273 | *sak*, *scn* (E) | - | *seh, sek+seq* | - | *blaZ* |
| K210N | Kimberley | Kalumburu (K) | 762 | t127 | *sak*, *scn* (E) | - | *seh, sek+seq* | - | *blaZ, ermC* |
| K45S | Kimberley | Kalumburu (K) | 762 | t273 | *sak*, *scn* (E) | - | *seh* | - | *blaZ, ermC* |
| K4T | Kimberley | Kalumburu (K) | 762 | t273 | - | - | *seh* | - | *blaZ* |
| W102T | Kimberley | Wangkatjungka (W) | 762 | t273 | *sak*, *scn* (E) | - | *seh* | - | *blaZ* |
| W104N | Kimberley | Wangkatjungka (W) | 762 | t273 | *sak*, *scn* (E) | - | *seh, sek+seq* | - | *blaZ* |
| W108S | Kimberley | Wangkatjungka (W) | 762 | t273 | *sak*, *scn* (E) | - | *seh, sek+seq* | - | *blaZ* |
| W110S | Kimberley | Wangkatjungka (W) | 762 | t273 | *sak*, *scn* (E) | - | *seh* | *eta* | *blaZ* |
| W149T | Kimberley | Wangkatjungka (W) | 762 | t273 | *sak*, *scn* (E) | - | *seh, sek+seq* | - | *blaZ* |
| W33T | Kimberley | Wangkatjungka (W) | 1 | t2478 | *sak*, *scn* (E) | - | *seh* | - | *blaZ, tet*(K) |
| W34T | Kimberley | Wangkatjungka (W) | 1 | t10349 | *sak*, *scn* (E) | - | *seh* | - | *blaZ* |
| W39S | Kimberley | Wangkatjungka (W) | 762 | t273 | *sak*, *scn* (E) | - | *seh, sek+seq* | - | *blaZ* |
| W3S | Kimberley | Wangkatjungka (W) | 762 | t273 | *sak*, *scn* (E) | - | *seh* | - | *blaZ* |
| W418S | Kimberley | Wangkatjungka (W) | 1 | t127 | *sea*, *sak*, *scn* (D) | - | *sea, seh, sek+seq* | - | *blaZ, mecA, ermC, fusC* |
| W5T | Kimberley | Wangkatjungka (W) | 762 | t273 | *sak*, *scn* (E) |  | *seh* | - | *blaZ* |
| W92S | Kimberley | Wangkatjungka (W) | 762 | t273 | *sak*, *scn* (E) | - | *seh, sek+seq* | - | *blaZ* |
| W95T | Kimberley | Wangkatjungka (W) | 762 | t273 | *sak*, *scn* (E) | - | *seh, sek+seq* | - | *blaZ* |
| W96S | Kimberley | Wangkatjungka (W) | 762 | t273 | *sak*, *scn* (E) | - | *seh, sek+seq* | - | *blaZ* |
| Y15S | Kimberley | Yiyili (Y) | 1 | t273 | *sea*, *sak*, *scn* (D) | - | *sea, seh, sek+seq* | - | *blaZ, fusC* |
| Y46N | Kimberley | Yiyili (Y) | 762 | t273 | *sak*, *scn* (E) | - | *seh, sek+seq* | - | *blaZ* |
| Y74T | Kimberley | Yiyili (Y) | 761 | t273 | *sak*, *scn* (E) | - | *seh, sek+seq* | - | *blaZ* |
| WL107T | Midwest | Wiluna (WL) | 1 | t127 | *sea*, *sak*, *scn* (D) | - | *sea, seh, sek+seq* | - | *blaZ, mecA, ermC, fusC* |
| WL10N-1 | Midwest | Wiluna (WL) | 1 | t127 | *sea*, *sak*, *scn* (D) | - | *sea, seh, sek+seq* | - | *blaZ, mecA, ermC, fusC, tet*(K) |
| WL10N-2 | Midwest | Wiluna (WL) | 1 | t127 | *sea*, *sak*, *scn* (D) | - | *sea, seh, sek+seq* | - | *blaZ, mecA, ermC, fusC, tet*(K) |
| WL22S-1 | Midwest | Wiluna (WL) | 1 | t127 | *sea*, *sak*, *scn* (D) | *lukF/S*-PV | *sea, seh, sek+seq* | - | *blaZ, mecA, fusC* |
| WL34S-2 | Midwest | Wiluna (WL) | 762 | t273 | *sak*, *scn* (E) | - | *-* | - | *blaZ* |
| WL55W-1 | Midwest | Wiluna (WL) | 1 | t11670 | *sak*, *scn* (E) | - | *seh* | - | *blaZ, mecA, fusC* |
| WL55W-2 | Midwest | Wiluna (WL) | 1 | t11670 | *sak*, *scn* (E) | - | *seh* | - | *blaZ, mecA, fusC* |
| WL93S-1 | Midwest | Wiluna (WL) | 1 | t127 | *sea*, *sak*, *scn* (D) | *lukF/S*-PV | *sea, sek+seq* | - | *blaZ, mecA, fusC* |
| WL96S-1 | Midwest | Wiluna (WL) | 1 | t559 | *sea*, *sak*, *scn* (D) | - | *sea, seh, sek+seq* | - | *mecA, fusC* |
| WL9S-1 | Midwest | Wiluna (WL) | 1 | t559 | *sea*, *sak*, *scn* (D) | - | *sea, seh, sek+seq* | - | *mecA, fusC* |
| J114N | Pilbara | Jigalong (J) | 1 | t127 | *sea*, *sak*, *scn* (D) | - | *sea, seh, sek+seq* | - | *blaZ, mecA, ermC, fusC* |
| J114S-1 | Pilbara | Jigalong (J) | 1 | t127 | *sea*, *sak*, *scn* (D) | - | *sea, seh, sek+seq* | - | *blaZ, mecA, ermC, fusC* |
| J162N | Pilbara | Jigalong (J) | 1 | t273 | *sea*, *sak*, *scn* (D) | - | *sea, seh, sek+seq* | - | *blaZ, mecA* |
| J183S | Pilbara | Jigalong (J) | 1 | t127 | *sea*, *sak*, *scn* (D) | - | *sea, seh, sek+seq* | - | *blaZ, mecA* |
| J183T | Pilbara | Jigalong (J) | 1 | t127 | *sea*, *sak*, *scn* (D) | - | *sea, seh, sek+seq* | - | *blaZ, mecA* |
| J183W | Pilbara | Jigalong (J) | 1 | t127 | *sea*, *sak*, *scn* (D) | - | *sea, seh, sek+seq* | - | *blaZ, mecA* |
| J197N-2 | Pilbara | Jigalong (J) | 1 | t127 | *sea*, *sak*, *scn* (D) | - | *sea, seh, sek+seq* | - | *blaZ, mecA, ermC* |
| J43N | Pilbara | Jigalong (J) | 1 | t127 | *sea*, *sak*, *scn* (D) | - | *sea, seh, sek+seq* | - | *blaZ, mecA, ermC, fusC* |
| J43S-1 | Pilbara | Jigalong (J) | 1 | t127 | *sea*, *sak*, *scn* (D) | - | *sea, seh, sek+seq* | - | *blaZ, mecA, ermC, fusC* |
| J83W | Pilbara | Jigalong (J) | 1 | t2478 | *sak*, *scn* (E) | - | *seh* | - | *blaZ* |
| J91T | Pilbara | Jigalong (J) | 1 | t127 | *sea*, *sak*, *scn* (D) | - | *sea, seh, sek+seq* | - | *blaZ, mecA, ermC* |

ST, sequence type; *spa*, staphylococcal protein A; IEC, immune evasion cluster; PVL, Panton-Valentine Leukocidin; AMR, antimicrobial resistance
